# Post-trial access and the new version of the Declaration of Helsinki

**Published:** 2013-12-30

**Authors:** Ricardo Palacios

**Affiliations:** Clinical Research and Development Manager, Division of Clinical Trial and Pharmacovigilance, Instituto Butantan São Paulo, Brazil, E-mail address: rpalacios@meridionalrd.com

In October 2013, the World Medical Association (WMA) approved the latest version of the Declaration of Helsinki (DoH) in Fortaleza, Brazil. Post-trial access of favorable interventions was again one of the critical issues raised during the meeting. The call to clinical research actors, other than physicians, in this discussion is not new, but this is the first time, after 49 years and nine amendments, when governments are requested to take responsibilities. 

The primary purpose of the DoH is the protection of human subjects involved in clinical research, but since the 2000 amendment, the WMA extended their concerns to what happens to the trial participants after the study. This issue, along with the use of placebo, was one of the key points that led to the withdrawal of the DoH as reference in the United States regulation. However, only the 2013 version acknowledges that the burden of providing post-trial access for those patients on continuous treatment is far beyond the investigator's scope. 

The delay between the end of a blind study and the unblinding could take several months. Meantime, the investigator is unaware if the participant received either control or experimental product. In case of therapeutic response, the investigator should provide to the participant the same treatment until the unblinding, even if it is the control product, i.e. placebo?.

A trial participant can get therapeutic response with an experimental product that during the unblinded analysis has failed. The risk benefit analysis on individual basis might be challenging if one considers that the clinical development can stop due to safety concerns, lack of efficacy or release of alternatives more advantageous than the investigational product. Before retaining a product still under development after a study, the physician should consider safety and efficacy information as well as therapeutic alternatives to take decisions on an individual participant, even if the patient has a favorable response with acceptable tolerance. Furthermore, product manufacturing can be discontinued, raising the need to switch the experimental product to a sustainable alternative.

When the study results are positive for the experimental group, there is a gap between the end of the study and the product registration by the national regulatory agency of the trial host country. The process might require results from additional studies and could take months to years to be available, if so, post-trial access means providing an unregistered treatment to former study subjects. Duration of the free supply of products after the study deserves special consideration, i.e. promising lifetime free product might become an undue inducement to participate in a trial in some clinical settings. 

In all the above-mentioned cases, the principal investigator alone cannot get the resources and information to warrant appropriate post-trial access, product supply and cumulative data on product development depends on the sponsor. Rollover studies have provided an alternative to keep experimental treatment for those patients in need; however, sometimes participation in those rollover studies is not feasible or desirable for some patients. The use of an unregistered product outside of a clinical trial environment requires a legal pathway. Adverse events vigilance has a key role to follow-up those patients not enrolled in rollover studies. Untoward reactions occurred on those patients might affect ongoing studies and clinical research centers running trials with the product should receive updates on safety and clinical response of those cases. Among the pharmacovigilance tasks there is also the preparation of risk minimization plans and the support to medication recall as new concerning information become available. Decisions regarding an individual subject should consider more than his or her clinical response to the treatment, but also the cumulative knowledge available and the sustainability of the proposed measures. Otherwise, medicine for former participants will be back to the times of the anecdotal evidence.

Once product succeeds and conquers first registration, the study subjects that where trials participants can face restrictions to access the treatment. Lack of registration in the participant's country and unaffordable price are among those barriers. Negotiation to get reassurance from pharmaceutical companies to have a product registered in a country at accessible price is roll of governments rather than investigators. National regulatory agencies can be in charge of this approach with the sponsor by the time the clinical development plan is discussed. 

The paragraph 34 of the DoH 2013 acknowledges that post-trial access would be possible only with concur of investigators, sponsors and host country governments. Incorporation of mechanisms to implement post-trial access into local regulations is a current challenge for all the regulatory and ethical authorities of study host countries. 

